# Integrating knowledge graphs and contextual patterns for legal concept alignment

**DOI:** 10.3389/frai.2026.1814303

**Published:** 2026-06-24

**Authors:** Jung-Shiuan Liou, Chih-Yao Yang, Yi-Shin Chen

**Affiliations:** 1 Intelligent Data Engineering and Applications Laboratory, International Intercollegiate Ph.D. Program, National Tsing Hua University, Hsinchu, Taiwan; 2 Intelligent Data Engineering and Applications Laboratory, Institute of Information Systems and Applications, National Tsing Hua University, Hsinchu, Taiwan; 3 Intelligent Data Engineering and Applications Laboratory, Department of Computer Science, National Tsing Hua University, Hsinchu, Taiwan

**Keywords:** AI-assisted legal concept alignment, contextual pattern, hierarchical structure, interpretability, knowledge graph

## Abstract

AI-assisted legal concept alignment, defined as identifying legal concepts that best correspond to a given fact description based on computational relevance, addresses the growing need for complex legal analysis that requires both semantic understanding and interpretability of judicial decision-making. This task presents three main challenges: reasoning-related contextual signals in judicial texts, complex hierarchical and cross-referential relationships among statutes, and highly similar wording in legal content. To address these challenges, this paper proposes a framework that integrates contextual pattern identification and law-centric knowledge graph construction, supported by hierarchical architectures and a multi-perspective scoring mechanism. Contextual pattern identification captures reasoning-related contextual signals within judicial texts. Law-centric knowledge graph and hierarchical architectures collectively address the problems of complex cross-referential relationships among legal elements and semantic ambiguity posed by closely related legal content. A multi-perspective scoring mechanism further evaluates the ranking quality of selected law articles. Experimental results show that the proposed framework captures both fine-grained details and broad semantic relations in legal content, achieving significant improvement in legal concept alignment compared with baseline methods. This paper delivers distinct value in pursuing ranking quality over pure alignment accuracy, capturing partial relevance among multiple law articles, and prioritizing model interpretability and reasoning traceability.

## 1 Introduction

Large language models (LLMs) have reshaped the legal landscape by improving the efficiency, consistency, and accessibility of legal services. Beyond fundamental tasks such as information retrieval, document classification, and case prediction, recent research has shifted toward more complex legal analysis that requires both semantic understanding and interpretability (Bhambhoria et al., 2022; Richmond et al., 2024; Almeida et al., 2025). As LLMs are increasingly deployed for legally sensitive tasks, including identifying applicable statutes or supporting legal reasoning, the transparency and explainability of model decisions have become critical (Cheong et al., 2024; Barman et al., 2024).

For criminal cases, one of the most important tasks during the judicial process is the legal qualification of facts, which is shaped by explicit and implicit legal knowledge, evidentiary assumptions, interpretive choices, and domain expertise. Determining applicable law articles, clauses, criminal charges, and penalties based on legal fact descriptions is critical for judges. AI-assisted legal concept alignment addresses this need by focusing on the identification of legal concepts—including law articles, clauses, and criminal charges—that correspond to a given fact description. Unlike conventional legal judgment prediction, which treats law article identification as mere semantic matching, AI-assisted legal concept alignment emphasizes ranking quality, partial relevance among law articles, and interpretability. This task is particularly important in Civil Law systems, where codified statutes serve as the primary basis for judicial decision-making and legal reasoning is expected to follow structured statutory references (Draper and Gillibrand, 2023).

However, accurate legal concept alignment presents three main challenges. First, judicial verdicts often embed legal reasoning implicitly within lengthy judicial texts. These verdicts contain extensive narrative information, including multiple and contradictory perspectives on the case, the motivations and attitudes of the defendant, the sequence of criminal events, and the validity of the collected evidence. Signals related to intent, causality, and legal stance are rarely described explicitly, making them difficult to extract using semantic similarity alone. Second, statutory law incorporates complex hierarchical structures and cross-references. Law articles frequently reference other articles or clauses through titles or codified identifiers; these layered references make it hard to determine which legal provisions are relevant. Third, fact descriptions and law articles often share highly similar wording but carry distinct legal implications. For example, “wrongful death” and “injury resulting in death” are distinct offenses but likely to feature similar fact descriptions. “Wrongful death” refers to death without harmful or lethal intent, while “injury resulting in death” involves death caused by intentional harm. These characteristics limit the effectiveness of deploying purely text-based or black-box models.

To address these challenges, this paper proposes an AI-assisted legal concept alignment framework that identifies contextual signals associated with legal reasoning and incorporates statutory cross-references. The framework integrates contextual pattern identification to distill reasoning-related signals from judicial verdicts and law-centric knowledge graphs to model hierarchical relationships and cross-references among statutes. Additionally, a hierarchical crime–law alignment architecture aggregates law articles based on their corresponding criminal offenses. Furthermore, a multi-granularity encoder and a multi-perspective scoring mechanism enhance the framework by jointly considering global semantic relations, fine-grained legal details, and exact statutory terminology.

Contextual signals associated with legal reasoning are captured by identifying contextual patterns. A contextual pattern refers to a set of contextual words appearing around a legal keyword that indicate its relevance. When such a pattern occurs, it signals an association with the corresponding legal concept. A metric named “Reasoning Degree” is introduced to quantify the strength of the relationship between contextual words and their associated legal keywords. Furthermore, through a specialized distillation process, we extract contextual patterns that capture reasoning-related contextual signals. This pre-processing step enhances reasoning-related signals and forms the foundation for subsequent legal concept alignment.

To address the challenges of hierarchical relationships and statutory cross-references, we construct a law-centric knowledge graph that models the relationships among law articles and criminal charges. Key elements—including law articles, clauses, and penalties—are extracted from the Criminal Code, and the resulting graph reflects the hierarchical relationships among legal concepts. Based on this knowledge graph, a hierarchical crime–law alignment architecture is designed, coupled with a multi-granularity encoder, to mitigate semantic confusion and ambiguity within legal texts. The encoder captures nuances at different levels of abstraction through text embeddings at the sentence, paragraph, and document levels. A scoring mechanism then assesses the semantic relationships between fact descriptions and law articles. Together, these components enhance the accuracy of legal concept alignment.

Our proposed framework delivers distinct value by prioritizing ranking quality over mere alignment accuracy, capturing partial relevance among multiple law articles, and maintaining model interpretability and reasoning traceability as core objectives. The contributions of this study are summarized below.

- An AI-assisted legal concept alignment framework is proposed to identify reasoning-related contextual signals and incorporate statutory cross-references, transcending the limitations of purely semantic matching and black-box prediction approaches.
- A contextual pattern identification method, along with a Reasoning Degree metric, is developed that distills contextual indicators associated with legal reasoning from judicial verdicts and enhances interpretability.
- A law-centric knowledge graph is constructed that captures hierarchical and cross-referential relationships among legal elements, enabling a more accurate interpretation of statutory dependencies.
- A hierarchical crime–law pair alignment is designed that reflects aspects of legal decision support and mitigates semantic ambiguity among closely related law articles.
- A multi-perspective scoring mechanism is introduced that integrates dense semantic similarity, fine-grained vector analysis, and lexical matching to improve ranking quality.

This paper is organized into five chapters. Chapter 1 outlines the motivations and challenges of AI-assisted legal concept alignment. Chapter 2 provides a comprehensive review of the relevant literature. Chapter 3 details the research methods, including contextual pattern identification, knowledge graph construction, hierarchical structure designs, and a multi-perspective scoring mechanism. Chapter 4 presents the experimental design and empirical findings, along with further discussions that validate the effectiveness of the proposed framework and highlight its limitations. Finally, Chapter 5 concludes the paper by summarizing the key contributions and exploring future directions.

## 2 Related work

In this section, an overview of alignment approaches is summarized in Table 1, followed by literature review on individual subsections.

**Table 1:** Summary of advantages and limitations of alignment methods (KGs : knowledge graphs).

Method	Advantages	Limitations
Translation-based entity alignment (Sun et al., 2017; Zhu et al., 2017, 2019)	Efficiently models one-to-one relations in KGs by translating a relationships from the source to the target entity.	Primarily focuses on local structures. Limited ability to capture complex relationships.
GNN-based entity alignment (Cao et al., 2019; Guo et al., 2018, 2021; Sang et al., 2023)	Designed to handle the complexity of heterogeneous KGs by capturing the neighborhood of an entity.	Limited ability to capture distant relationship semantics and relies on high-quality seeds. Requires hyperparameter tuning for optimal performance.
Reasoning-based entity alignment (Li et al., 2023; Meilicke et al., 2024; Das et al., 2017; Tan et al., 2023; Akhtar et al., 2022, 2025)	Performs reasoning and learns relationship semantics in KGs. Often achieves higher accuracy by resolving contradictions.	Logic-based reasoning may face scalability bottlenecks. Requires well-defined relationship explanations.
Feature-based identity linkage (Zhou et al., 2017; Li et al., 2018; Zhong et al., 2018)	Explicitly defines and extracts features from raw data, making them explainable.	Relies on manually designed attributes and relationships. Limited by the quality and relevance of the chosen features.
Embedding-based identity linkage (Yang et al., 2022; Zheng et al., 2022; Tang et al., 2023; Xiong et al., 2023; Peng et al., 2025)	Captures latent relationship in user behavior or network structures. Performs semantic robustness.	Hard to explain due to black-box nature. Difficult in handling noisy or unstructured data.
ML-based and DL-based legal prediction (Hu et al., 2018; Gan et al., 2021; Zhao et al., 2022)	Designed to effectively extract features and generate predictions. Provides data-driven insights.	Operates in black-box systems and lack of understanding of the reasoning process. Inherits systematic biases of raw data.

### 2.1 Entity alignment

Entity alignment (EA) refers to identifying entity pairs that point to the same real-world objects across multiple knowledge graphs. EA depends mainly upon translation-based, GNN-based, and reasoning-based approaches. GNN-based models (Cao et al., 2019; Guo et al., 2021; Sang et al., 2023) rely on graph-based frameworks to learn both entity and relationship representations in knowledge graphs. However, this approach has difficulty in handling complex relationships and scalability in large knowledge graphs. To address these limitations, RSA (Akhtar et al., 2022) leverages neighboring entities and relationship semantics context to generate embeddings for each entity in knowledge graphs. Reasoning-based models learn relationship semantics within knowledge graphs to perform logical reasoning. Models like TransO (Li et al., 2023) and AnyBURL (Meilicke et al., 2024) extract logical rules to perform ontological reasoning in multilingual knowledge graphs. MINERVA (Das et al., 2017) applies reinforcement learning to model logical reasoning for question-answering (QA) and inference tasks.

Based on an iterative framework, PRASEMap (Qi et al., 2021) combines probabilistic reasoning and semantic embedding modules to compute alignment, while CLRN (Tan et al., 2023) integrates QA and EA models to extract potential alignment triples. MRAEA (Mao et al., 2020) aligns knowledge graphs using learned entities and relation embeddings. By incorporating small language models (SLMs) and LLMs, HLMEA (Jin et al., 2025) utilizes an iterative self-training process that allows SLMs to distill knowledge from LLMs and enhance EA performance. Additionally, AKR (Akhtar et al., 2025) integrates centrality calculation and relational semantics reasoning to connect distant entities in multilingual knowledge graphs. EA approaches demonstrating logic and interpretability for aligning entities illustrate a potential path toward aligning knowledge graphs with explainability.

### 2.2 Identity linkage

Identity linkage refers to identifying user accounts across diverse social networks, even without explicit interconnections. As described in the survey by Senette et al. (2024), this can be formulated as a classification task or a network alignment task. In feature-based strategies, models like FRUI-P (Zhou et al., 2017) and the Authority-Trustworthiness Analysis model (Li et al., 2018) extract friend features and evaluate the similarities of potentially identical users by assessing the authority and reliability of a user's friends. CoLink (Zhong et al., 2018) iteratively leverages a co-training algorithm that includes an attribute-based model and a relationship-based model. BSNA (Yuan et al., 2021) utilizes backpropagation neural network to realize the mapping between two social network username vectors and changes the classification into a mapping problem.

In embedding-based strategies, (Yang et al., 2022) developed an anchor link prediction method based on multiple consistency through network representation learning. JORA (Zheng et al., 2022) jointly employs representation learning to preserve similarities within networks and alignment learning to align across networks. CPUM (Tang et al., 2023) models user attributes and network topology, capturing alignment through both topology and attribute consistency. DSANE (Xiong et al., 2023) enhances alignment by adding relevant edges between seed user pairs across networks and removing less useful nodes. GSMUA (Peng et al., 2025) applies a gradient semantic model by categorizing user profiles into multi-level gradients to achieve complete semantic representations of user attributes. For aligning user identities across diverse networks, feature-based approaches provide interpretable insights, while embedding-based approaches enhance performance by utilizing additional features. This suggests a combined method that can improve alignment quality while maintaining interpretability.

### 2.3 Legal knowledge extraction

The challenges of extracting legal knowledge lie in navigating the intricate structures of legal documents and developing effective extraction strategies (Moens et al., 1999; Rabelo et al., 2020). Traditional statistical (Rose et al., 2010; Campos et al., 2020) and graph-based approaches (Mihalcea and Tarau, 2004; Wan and Xiao, 2008), as well as machine learning methods (Grootendorst, 2022; Priyanshu and Vijay, 2022), have been extensively explored in the literature. Focusing on concept extraction, ASKE (Castano et al., 2024) employs an iterative extraction process using context-aware embeddings and zero-shot learning. With the emergence of LLMs, transformer-based models have demonstrated potential for knowledge extraction without domain-specific training data. However, they still face challenges such as generating factual errors, producing hallucinated details, and misinterpreting linguistic nuances (Siino and Tinnirello, 2024; Haque and Singh, 2025). Effectively processing judicial texts requires an extraction strategy that captures conceptual and contextual information while ensuring factual integrity. This suggests a combination of classical approaches and hierarchical designs.

### 2.4 Legal knowledge graph

Studies on the construction of legal knowledge graphs (KGs) primarily focus on two aspects: classification and relation extraction. (Biao et al., 2021) optimized a Bidirectional Gated Recurrent Unit (Bi-GRU) model with an improved cross-entropy loss function, to extract attribute relationships from unstructured legal texts. (Zhou et al., 2024) applied pre-trained language models for entity recognition and a multi-task semantic relationship extraction model. OKE tools (Sovrano et al., 2020) analyze the grammatical dependencies of legal content to construct KGs for QA tasks. Similarly, (Zhu et al., 2022) developed a public prosecutorial knowledge system by combining Bidirectional Long-Short-Term Memory (Bi-LSTM) networks with Conditional Random Fields (CRFs). Legal-LM (Shi et al., 2024), a KG-enhanced large language model, compiles a vast array of raw legal texts and integrates them into structured data formats. These implementations demonstrate that knowledge graphs can retrieve legal information efficiently. Furthermore, this suggests a viable path for addressing the complexity and cross-referential relationships of legal statutes by leveraging knowledge graphs.

### 2.5 LLMs in legal applications

According to the survey by (Siino 2025), LLMs have been widely applied to various legal tasks, including document classification, knowledge extraction, summarization, case and article retrieval, document automation, named entity recognition, and semantic similarity. Additionally, several studies focus on optimizing prompting techniques to improve LLMs' performance (Trautmann et al., 2022; Yu et al., 2022; Blair-Stanek et al., 2023; Gupta et al., 2025). (Nguyen et al., 2025) integrated rule-based, abductive, and case-based reasoning with LLMs to provide adaptable legal analysis. To enhance the legal reasoning and reliability of LLMs, recent literature introduces strategies such as fine-tuning (Yue et al., 2023), customized pre-processing and in-context learning (Shu et al., 2024), and Mixture-of-Experts (MoEs) architectures combined with a multi-agent system (Cui et al., 2024).

Other research focuses on evaluating LLM capabilities at different cognitive levels (Fei et al., 2023), incorporating citations (Zhang et al., 2025), mimicking the reasoning of legal practitioners (Enguehard et al., 2025), and assessing multi-task performance (Li et al., 2025). Meanwhile, Siino and Tinnirello (2024) employed LLMs with few-shot learning and prompt engineering to detect GPT hallucination. Although LLMs present a promising future for legal practices, the deployment has raised concerns regarding data sovereignty, algorithmic transparency, judicial practices, and reliability (Padiu et al., 2024; Doyle and Tucker, 2025). Recently, the autonomy of agentic AI further poses legal and ethical challenges to existing laws and regulations (Bowen, 2025; Reed, 2025; Murray, 2025).

## 3 Methodology

### 3.1 Overview

This paper proposes a framework for identifying legal concepts—including applicable law articles, clauses, and criminal charges—that align with a given fact description. To achieve accurate alignment, the model must address three main challenges. The first is the reasoning-related contextual signals within judicial verdicts, which contains intent, causality, and legal stance. The second involves the complex hierarchical and cross-referential relationships within legal statutes, where law articles frequently cite one another through titles or codified identifiers. The third challenge concerns the high degree of semantic similarity between fact descriptions and law articles, which often results in ambiguity. Finally, to measure alignment quality, we use Precision of the predicted legal concepts as the primary evaluation metric. Based on this approach, the problem is defined as follows.

** Definition 1**. (Problem Definition) Given a fact description of a test case *f*_test_ in a training dataset $D={\left\{\left(f_{z},y_{z}\right)\right\}}_{z=1}^{q}$ of size q, where *f*_*z*_ denotes a fact description incorporating its associated legal keywords, *y*_*z*_ is the corresponding categorical variable from *Y* = {*y*_1_, …, *y*_*t*_}, the objective is to train a model *f* that identifies the legal concepts *L*, including the law articles, clauses, and criminal charges, aligned with the given fact description *f*_test_. The model generates


$$ŷ=f\left(f_{\text{test}},L\right)$$

where ŷ⊆*Y*, *L* = {*L*_1_, …, *L*_*m*_} denotes the associated legal concepts.

The methodology begins by pre-processing judicial verdicts to capture reasoning-related contextual signals. Using these enhanced signals, the portions of fact descriptions that indicate legal stances are extracted, a step essential for ensuring accurate legal concept alignment. Subsequently, a knowledge graph is constructed from legal elements—including law articles, clauses, and penalties—extracted from the Criminal Code. Legal elements corresponding to specific criminal offenses are then aggregated. By combining these knowledge graphs with crime-perspective aggregation, hierarchical crime-law pairs are formulated. Additionally, a multi-granularity encoder is employed to capture semantic nuances at the sentence, paragraph, and document levels. Finally, a multi-perspective scoring mechanism is developed to prioritize the semantic relationships between fact descriptions and law articles. Together, these components enhance the alignment accuracy of applicable law articles, clauses, and criminal charges while maintaining interpretability. The general procedure is described in Algorithm 1.

Algorithm 1 AI-assisted legal concept alignment.

Input:  Fact descriptions *F*, Law articles *L*
Output:  Alignment result *y* incorporates associated legal concepts
1:  Initialize: Extract legal elements (law articles, clauses, penalties) from *L*.
2:  Perform elements completion and construct law-centric knowledge graphs *G*.
3:  Aggregate *G* via crime-perspective aggregation to form hierarchical crime-law pairs *H*.
4:  Encode *F* through multi-granularity encoder to obtain fact embeddings *E*_*f*_.
5:  for each crime-law pair in *H* **do**
6:   Perform pairwise comparison between *E*_*f*_ and crime embeddings.
7:   if similarity score exceeds selection threshold **then**
8:   Add law to candidate set *C* and retrieve candidate sub-graph *G*_*cand*_.
9:   end **if**
10:  end **for**
11:  for each candidate *c*∈*C* **do**
12:   Calculate dense score *S*_*dense*_, multi-vector score *S*_*multi*_, and lexical score *S*_*lexical*_.
13:   Integrate scores to generate the unified Inter Score *S*_*final*_.
14:  end **for**
15:  Compute law inter scoring to evaluate dependencies between candidate law articles.
16:  Perform crime-law selection based on *S*_*final*_ and cross-law relationships.
17:  Generate alignment results *y*.
18:  return *y*

**Figure 1:**
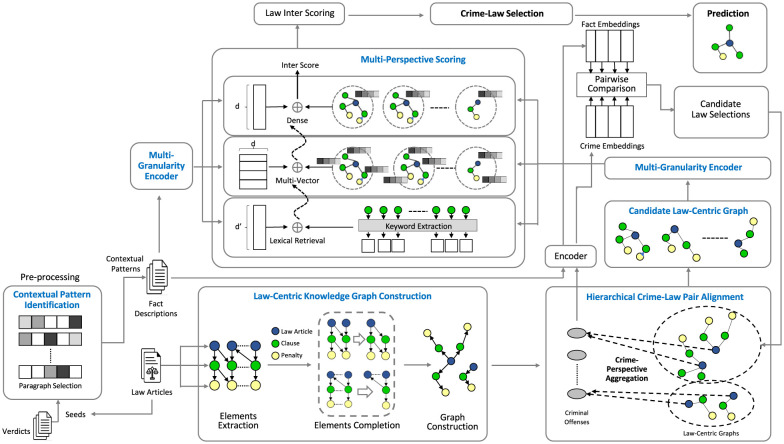
The overall framework.

The overall framework, illustrated in Figure 1, consists of six components.

- Contextual Pattern Identification: Capture reasoning-related contextual signals from judicial verdicts by identifying contextual patterns that correspond to relevant legal keywords.
- Law-Centric Knowledge Graph Construction: Construct a knowledge graph using legal elements, including law articles, clauses, and penalties, extracted from the Criminal Code.
- Hierarchical Crime-Law Pair Alignment: Aggregate law articles by individual criminal offense to establish hierarchical crime-law pairs aligned with specific offenses.
- Pairwise Comparison: Map fact descriptions and crimes into an embedding space and compare these representations to produce a set of candidate law selections.
- Multi-Granularity Encoder: Generate embeddings at the sentence, paragraph, and document levels to precisely depict the semantic abstractions of legal texts.
- Multi-Perspective Scoring: Prioritize candidates by integrating dense representations, multi-vector analysis, and lexical retrieval between fact descriptions and law articles.

### 3.2 Contextual pattern identification

**Figure 2:**
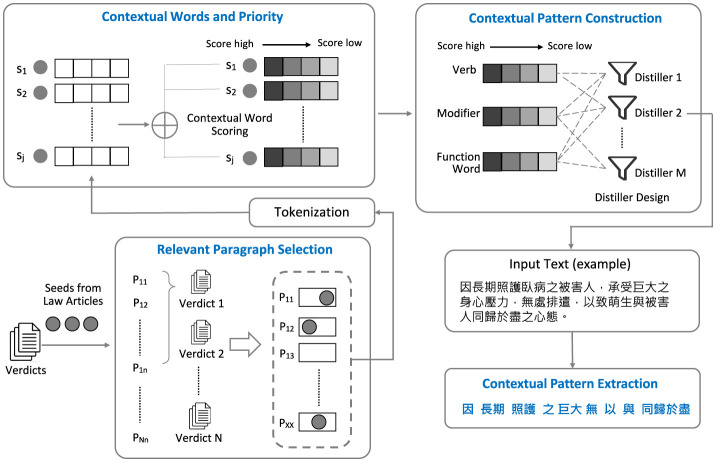
Contextual pattern identification.

Judicial verdicts encompass extensive narrative information, including multiple and conflicting perspectives, defendant motivations and attitudes, chronological sequences of criminal events, and the validity of the collected evidence. Due to their length and complexity, these verdicts frequently embed legal reasoning implicitly. Contextual patterns identification aims to capture reasoning-related contextual signals within fact descriptions. Specifically, when a cluster of contextual words appears in proximity to a legal keyword, it constitutes a contextual pattern that signals a semantic association with the corresponding legal concept. This pre-processing step serves as the foundation for subsequent legal concept alignment, as illustrated in Figure 2. The process is detailed below.

Step 3.2.1 (Relevant Paragraph Selection) Typically, a judicial verdict contains multiple paragraphs for various purposes. To extract relevant paragraphs, a seed selection approach is introduced, which is inspired by the bootstrap sampling technique. The selected seeds consist of legal keywords found in related law articles. The paragraphs containing these seeds are extracted to capture reasoning-related contextual signals.

Step 3.2.2 (Contextual Words and Priority) Relevant paragraphs are tokenized to generate contextual words, which are aggregated based on individual seeds. The challenge lies in prioritizing these words according to their relationships with the associated seeds. Words that frequently appear in most paragraphs may be too general for effective discrimination. Conversely, words with lower frequency but strong relevance are more informative. To assess word distinguishability among different seeds, a metric called Reasoning Degree *rd* is introduced to evaluate the association between contextual words and their corresponding seeds. The metric *rd* is defined in Equation 1.


$$\begin{array}{l} rd\left(\text{cw},s_{j}\right)=cwf\left(\text{cw},s_{j}\right)\times ipf\left(\text{cw},s_{j}\right)\times dd\left(\text{cw}\right) \end{array}$$ (1)

Where *cwf*(cw, *s*_*j*_) denotes the frequency of the contextual word *cw* associated with the seed *s*_*j*_∈*S*; *ipf*(cw, *s*_*j*_) denotes Inverse Paragraph Frequency, which measures the prevalence of *cw* across all paragraphs under a given seed; and *dd*(cw) denotes Discrimination Degree, which assesses the uniqueness of *cw* in relation to that seed. Using this *rd* metric, contextual words are prioritized based on the strength of their relationships with their associated seeds. A higher-ranking contextual word indicates greater distinguishability for that seed.

Compared to pure text matching, this frequency-based metric *rd* demonstrates its superiority in three ways. First, it uses seeds representing legal concepts as anchor words to navigate reasoning-related contextual signals. Second, by measuring uniqueness, it rewards specific contextual words instead of treating all terms equally. This helps filter out noise that is common in judicial texts. Third, it assesses the prevalence across all paragraphs, which ensures the consistency of the identified reasoning. Considering *rd* is tied to seed association and paragraph level, it is more robust in handling topic draft and long documents.

Step 3.2.3 (Contextual Pattern Construction) Contextual words with different POS tags may yield varying values depending on their semantic roles. Including all POS tags introduces noise and reduces the performance of the model. To this end, only *Verbs, Modifiers*, and *Function Words* are retained, as specified in Equation 2.


$$\begin{array}{l} CW_{s_{j}}=\left\{V_{s_{j}}\cup M_{s_{j}}\cup F_{s_{j}}\right\} \end{array}$$ (2)

where *CW*_*s*_*j*__ denotes contextual words under the seed *s*_*j*_∈*S*, *V*_*s*_*j*__, *M*_*s*_*j*__, and *F*_*s*_*j*__ denote contextual words categorized as verbs, modifiers, and function words, respectively.

Furthermore, we examine various combinations of contextual words through a specialized distillation process formulated in Equation 3. This step identifies informative contextual patterns that capture reasoning-related contextual signals while minimizing the impact of noise.


$$\begin{array}{l} D_{v,m,f}=\underset{L\in \left\{V,M,F\right\}}{⋃}CP_{top}^{L}. \end{array}$$ (3)

where *D*_*v, m, f*_ denotes a distiller design with varying parameters, $CP_{top}^{L}$ denotes contextual patterns incorporating the top percentages of contextual words based on their *rd* scores.

Through this pre-processing step, reasoning-related contextual signals are captured by identifying contextual patterns. Legal stance inference, which determines whether a sentence strengthens, weakens, or remains neutral with respect to a criminal offense, is performed to evaluate the usefulness of these contextual patterns.

### 3.3 Law-centric knowledge graph construction

A law-centric knowledge graph is constructed to capture the relationships between law articles and criminal charges. Key elements, including law articles, clauses, and penalties, are extracted from the Criminal Code, and the resulting graph reflects the hierarchical relationships among legal concepts. The extraction rules are specified below.

1. If a clause contains the term ‘preceding clause' (前項), replace it with the text of that clause.
2. If a clause contains the term ‘preceding law article' (前條), replace it with the text of that law article.
3. If a clause contains the term ‘one of the following' (下列之一), replace it with the text of that corresponding clause.
4. If a clause contains the term ‘specific citations in the format' (第[一二三四五六七八九十]+條第[一二三四五六七八九十]+項), replace it with the text of that specific clause in the format.
5. The terms ‘preceding clause' (前項), ‘preceding law article' (前條), ‘one of the following' (下列之一), and ‘specific citations in the format' (第[一二三四五六七八九十]+條第[一二三四五六七八九十]+項) are utilized as seed terms. Clause embeddings are compared with these seed embeddings. If a clause embedding demonstrates high similarity that meets the matching threshold to a seed, the clause is replaced with the text of that corresponding citation.

**Figure 3:**
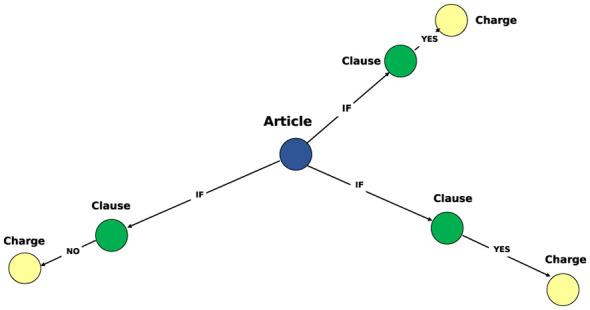
An example knowledge graph.

The graph structure is enhanced by incorporating additional contextual information and relationships among legal clauses through the combination of manual extraction rules and an embedding-based approach. The former addresses the challenge of statutory cross-references, while the latter extends the model's adaptability to dynamic legal corpora. In this paper, the manual extraction method is primarily applied to process the Criminal Code, where the formats of law articles are strictly defined. An example of the knowledge graph is presented in Figure 3. Each node represents a law article, clause, or penalty, and each edge represents the relationship between two nodes, such as cross-references or hierarchical connections. The label on each edge denotes the nature of the relationship, such as a reference to a specific clause. The formal definition of the knowledge graph is provided below.

** Definition 2**. (Knowledge Graph Construction) Given a knowledge graph *G* = (*V, E*) that consists of a set of vertices *V* and a set of edges *E*, where each vertex *v*∈*V* represents either a law article, clause, or penalty, and each edge *e* = (*v*_*i*_, *v*_*j*_)∈*E* describes the relationship from vertex *v*_*i*_ to vertex *v*_*j*_. Each edge *e* is labeled with *l*(*e*), which indicates the type of reference (e.g., specific clause, penalty).

### 3.4 Hierarchical crime-law pair alignment

Through constructed law-centric knowledge graphs, legal elements are aggregated from a crime-oriented semantic perspective. Inspired by the alignment methods investigated earlier, in this task, a hierarchical law structure is designed to correspond to a specific offense. Relational Graph Convolutional Networks (R-GCNs) (Schlichtkrull et al., 2018) are utilized to generate representations of knowledge graphs, ensuring offense features propagate bi-directionally through the hierarchy. Meanwhile, individual offenses are treated as nodes within a separate result graph. Based on their structural similarities, a knowledge graph is assigned an offense label. Subsequently, knowledge graphs corresponding to a specific criminal offense are combined into a semantic cluster. This crime-centric aggregation reshapes knowledge graphs to capture the hierarchical and semantic relationships between individual offenses and their associated legal elements, ultimately forming hierarchical crime–law pairs.

** Definition 3**. (Hierarchical Crime-Law Pair Alignment) Given a set of criminal offenses *C* = {*C*_1_, *C*_2_, …, *C*_*n*_}, for each offense *C*_*i*_, the related law articles, clauses, and penalties that correspond to *C*_*i*_ are combined into a semantic cluster *H*_*i*_. The crime-based clusters form the hierarchical crime-law pairs by the following steps.

1. *Generate representations of law-centric knowledge graphs based on aggregated legal elements*.
2. *Each knowledge graph corresponds to a specific criminal offense C_i_*.
3. *Combine knowledge graphs with the same offense label to form a semantic cluster H_i_, representing the criminal offense C_i_ with its associated law articles, clauses, and penalties*.

### 3.5 Multi-perspective scoring

In the construction of law-centric knowledge graphs, complete legal elements are incorporated by addressing the challenge of statutory cross-references. Following crime–law pair alignment, a higher-level legal context is established through crime-centric aggregation. This complete legal context is then encoded into a comprehensive textual representation. To mitigate semantic ambiguity and confusion, a multi-granularity encoder is introduced to capture legal nuances at the sentence, paragraph, and document levels.

Subsequently, both fact descriptions and aggregated law articles are processed through a multi-perspective scoring mechanism to assess their semantic relationships. By integrating feature-based and embedding-based strategies, this mechanism generates three complementary representations. First, dense representations compare global semantic similarity between fact descriptions and law articles. Second, token-level multi-vector analysis captures fine-grained matching of legal elements. Third, lexical retrieval evaluates the precision of legal terminology, because in legal contexts a lexical mismatch can invalidate otherwise semantically similar reasoning. The multi-perspective scoring mechanism involves the following steps.

Step 3.5.1 (Dense Representation) Dense representations of both fact descriptions and law articles are generated using the multi-granularity encoder. Let *e*_*f*_ represent the embedding of a fact description *f*, and let *e*_*l*_ denote the embedding of a law article *L*_*i*_. The similarity score *s*_dense_ is computed as follows as Equation 4.


$$\begin{array}{l} s_{\text{dense}}=\text{cosine}\left(e_{f},e_{l}\right) \end{array}$$ (4)

Step 3.5.2 (Multi-Vector Analysis) To capture the relevance between fact descriptions and law articles, we utilize multi-vector representations to encompass various semantic aspects. These embeddings are compared at a granular level. Let {*e*_*f*1_, *e*_*f*2_, …, *e*_*fk*_} and {*e*_*l*1_, *e*_*l*2_, …, *e*_*lk*_} denote the sets of multi-vector embeddings for the fact description and law article, respectively. Each vector in these sets corresponds to a distinct semantic component. The multi-vector similarity score *s*_multi_ is calculated by aggregating the scores of the corresponding vectors, as Equation 5


$$\begin{array}{l} s_{\text{multi}}=\frac{1}{k}\overset{k}{\underset{i=1}{\sum }}\text{cosine}\left(e_{fi},e_{li}\right) \end{array}$$ (5)

Step 3.5.3 (Lexical Retrieval) Dense embeddings are transformed into sparse representations to perform lexical retrieval. This step involves computing the importance of each term based on its embedding. Let *w*(*t*) and *w*_*l*_(*t*) denote the weights of each term *t* in the fact description and law article, respectively. The lexical similarity score *s*_lexical_ is computed based on the overlap and significance of the terms extracted from the fact description and law article, as Equation 6


$$\begin{array}{l} s_{\text{lexical}}=\underset{t\in f\cap L_{i}}{\sum }w\left(t\right)\cdot w_{l}\left(t\right) \end{array}$$ (6)

Finally, the integrated score aggregates the dense representations, multi-vector analysis, and lexical retrieval, as defined below.

** Definition 4**. (Multi-Perspective Scoring) The multi-perspective score *s*_final_ is calculated by combining the similarity scores of dense representation *s*_dense_, multi-vector analysis *s*_multi_, and lexical retrieval *s*_lexical_, as Equation 7.


$$\begin{array}{l} s_{\text{final}}=\alpha s_{\text{dense}}+\beta s_{\text{multi}}+\gamma s_{\text{lexical}} \end{array}$$ (7)

where α, β, and γ are the weights that balance the contributions from the different scores. Specifically, α reflects the importance of dense semantics similarity, β reflects the importance of relationships, and γ reflects the importance of keyword matching.

The multi-perspective scoring mechanism provides a comprehensive evaluation of the alignment between fact descriptions and law articles. Integrated with the aforementioned components, the proposed framework enhances alignment accuracy while maintaining interpretability.

## 4 Experiment and results

### 4.1 Experimental setup

#### 4.1.1 Dataset

The dataset used in this paper comprises judicial verdicts and law articles. In the pre-processing step, homicide cases were chosen because they provide a wide spectrum of legal reasoning, ranging from ‘not guilty' to the ‘death penalty'. Additionally, a subset of 20% of the homicide cases was manually annotated by experienced lawyers to serve as the ground truth for legal stances. In subsequent steps, additional criminal offenses were incorporated: offenses against public safety, offenses of counterfeiting currency, offenses against reputation and credit, and offenses of escape. A total of 5,976 judicial verdicts were collected, ranging from minor infractions to serious criminal offenses, to ensure diversity among offense categories. Every judicial verdict incorporates a sentencing outcome—including the criminal charges, applicable law articles, and the term of penalty—serving as the validation for legal concept alignment. The statistics in Table 2 show that, on average, each case cites 7.66 law articles and involves 2.64 types of criminal offenses. This illustrates the difficulty of correctly identifying the legal concepts that align with a given fact description.

**Table 2:** Statistics of the dataset.

Statistics	Value
Total number of entries	5,976
Average number of applicable law articles per case	7.66
Average number of aggregated offenses per case	2.64

#### 4.1.2 Baseline

In the proposed framework, multiple methods are developed to extract semantic features ranging from the word level to the document level. The following baseline models are employed to evaluate the performance and robustness of the framework.

- TF-IDF: Term Frequency-Inverse Document Frequency is a statistical metric used to evaluate the importance of a word within a document relative to its frequency across a corpus.
- BM25 (Robertson and Zaragoza, 2009): Best Matching 25, a ranking function derived from the TF-IDF, is a probabilistic model employed by search engines to rank the relevance of documents based on a given search query.
- BERT (Devlin et al., 2019): Bidirectional Encoder Representations from Transformers is a pre-trained language model designed to be fine-tuned for a wide range of downstream NLP tasks.
- RoBERTa (Liu, 2019): Robustly Optimized BERT Pre-training Approach, which improves upon BERT by utilizing larger mini-batches and more extensive training data, achieves superior performance across various NLP benchmarks.
- Longformer (Beltagy et al., 2020): The Long-Document Transformer is designed to process extensive documents by utilizing a combination of local and global attention mechanisms to handle long-range context efficiently.
- Lawformer (Xiao et al., 2021): A Longformer-based language model pre-trained on legal corpora, is designed specifically to capture the nuances of legal language and structure. It is fine-tuned for tasks pertaining to legal prediction.
- BGE M3 (Chen et al., 2024): The embedding model, characterized by its multi-functional, multilingual, and multi-granular capabilities, is designed to process long token sequences by efficiently encoding and aggregating information from legal corpora.
- TAIDE (Science and, NSTC): Trustworthy AI Dialogue Engine, a localized large language model based on Gemma-3-12b, is fine-tuned on specific datasets to ensure high proficiency in Chinese and local cultural contexts. We further fine-tuned it with LoRA (Low-ranked Adaptation).

#### 4.1.3 Evaluation method

The value of our proposed framework is demonstrated through ranking quality, the handling of law articles with partial relevance, and model interpretability. Given these characteristics, traditional metrics such as accuracy and F1 score are insufficient. Therefore, the following evaluation metrics are utilized to assess the precision and quality of legal concept alignment.

- Priority Ranking Recall: This metric measures how effectively the model retrieves relevant law articles within the top *K* selections. It indicates the model's ability to identify all true law articles as early as possible in the ranked list. The metric is defined as Equation 8:
$$\begin{array}{l} \text{Priority Ranking Recall}=\frac{N_{true\text{\_}total}}{R_{max}} \end{array}$$ (8)
  where *N*_*true*_*total*_ represents the total number of true law articles, and *R*_*max*_ denotes the maximum ranking position of the true law articles in the top K selections.
- Rank Position Cost: This metric evaluates the cost associated with identifying all relevant law articles within the ranked list. It reflects how far down the list a user must search to retrieve all true law articles. The metric is defined as Equation 9:
$$\begin{array}{l} \text{Rank Position Cost}=\frac{R_{max}}{N_{law\text{\_}total}} \end{array}$$ (9)
  where *R*_*max*_ is the maximum ranking position of the true law articles, and *N*_*law*_*total*_ is the total number of law articles considered.
- Precision@K: This metric measures the precision of the top *K* selected law articles. It is computed as the number of true law articles within the top *K* selections divided by *K*. The metric is defined as follows Equation 10:
$$\begin{array}{l} \text{Precision@K}=\frac{N_{true@K}}{K} \end{array}$$ (10)
  where *N*_*true@K*_ is the number of true law articles within the top *K* selections and *K* represents the rank threshold. In this study, Precision@2, Precision@6, and Precision@10 are examined to provide a comprehensive evaluation of the model's precision across different rank levels.
- Weighted Score: To aggregate the results from multiple evaluation metrics, we utilize a weighted scoring system. This system combines the contributions of dense representations, multi-vector analysis, and lexical retrieval. Let *S*_*d*_, *S*_*m*_, and *S*_*m*_ denote the Dense, Multi-vector, and Lexical scores, respectively. The weighted score is defined as Equation 11:
$$\begin{array}{l} \text{Weighted Score}=\alpha S_{d}+\beta S_{m}+\gamma S_{l} \end{array}$$ (11)
  where α, β, and γ are the weights assigned to these scores, respectively.

#### 4.2 Experimental results and discussion

#### 4.2.1 Contextual pattern identification

The pre-processing step aims to capture reasoning-related contextual signals through identifying contextual patterns. These enhanced signals of legal reasoning within fact descriptions form the foundation for subsequent legal concept alignment. The accuracy of legal stance inference is then used to verify the effectiveness of these contextual patterns.

Compared to the traditional TF-IDF method, which primarily captures generic high-frequency terms, our proposed metric—Reasoning Degree—identifies unique and discriminative contextual words, as shown in Table 3. For instance, the top three keywords ranked by TF-IDF—“defendant,” “plaintiff,” and “victim”—are generic legal terms that frequently appear across various cases, offering limited contextual distinction. In contrast, the top three modifiers identified by our proposed method—“fiery,” “furious,” and “humble”—explicitly convey intent and emotional stance, making them valuable indicators for reasoning. This highlights the effectiveness of our proposed metric over TF-IDF in capturing meaningful, case-specific expressions of intent.

**Table 3:** Top 10 keywords of “Intention” (^*^: Appendix B).

Top K	TF-IDF	$CP_{top}^{Verb^{*}}$	$CP_{top}^{Modifier^{*}}$	$CP_{top}^{Function^{*}}$
				
1	Defendant	Explode	Fiery	To
2	Plaintiff	Expel	Furious	If
3	Victim	Leak	Humble	This
4	Court	Fall	Another	Whether
5	Behavior	Tow	Firm	And
6	Homicide	Throw	Dynamic	Even if
7	Witness	Inject	Controversial	Though
8	Harm	Kick	Turn Right	Even Though
9	Intention	Congest	Turn left	These
10	Then	Extend	Forward	As in

For stance inference, BERT, RoBERTa, and TAIDE are adopted as baseline models, relying solely on raw input texts. Among these baselines, TAIDE, based on Gemma-3, significantly outperforms BERT and RoBERTa by 7–8%, because it is tailored to meet specific linguistic and regulatory requirements. Table 4 shows that, when using contextual patterns only, our method achieves higher accuracy than BERT (with 2.93% improvement) and RoBERTa (with 3.37% improvement); while for TAIDE, the accuracy is comparable or slightly lower than the baseline. It indicates that our method effectively capture reasoning-related contextual signals even with partial input texts.

**Table 4:** Performance comparison using different contextual pattern configurations (^*^: Appendix B).

Method	Baseline	$CP_{top}^{Verb^{*}}$	$CP_{top}^{Modifier^{*}}$	$CP_{top}^{Function^{*}}$	Accuracy	Improvement
Baseline	BERT	–	–	–	84.60%	–
Contextual patterns	BERT	15	20	30	86.95%	2.35%
		20	20	30	87.22%	2.62%
		30	30	40	87.53%	2.93%
		40	40	50	87.45%	2.85%
Baseline	RoBERTa	–	–	–	85.62%	–
Contextual patterns	RoBERTa	15	20	30	86.74%	1.12%
		20	20	30	88.21%	2.59%
		30	30	40	87.16%	1.54%
		40	40	50	88.99%	3.37%
Baseline	TAIDE	–	–	–	92.35%	–
Contextual patterns	TAIDE	15	20	30	91.60%	-0.75%
		20	20	30	92.16%	–0.19%
		30	30	40	89.67%	–2.68%
		40	40	50	91.81%	–0.54%

Combining full text with contextual patterns as input enhances model performance across various distillation designs. Table 5 shows that the most effective configuration achieves 90.18% accuracy with BERT, 91.24% with RoBERTa, and 92.64% with TAIDE. These varying results across baseline models suggest two primary observations. First, for BERT and RoBERTa, which are not pre-trained on domain-specific corpora, our method provides more concise data representations and substantially improves their ability to capture semantic relationships. Second, for TAIDE, which is explicitly fine-tuned on Chinese content and local cultural contexts, our method achieves a comparable level of performance on full text and elevates accuracy less impressively. In addition, because TAIDE was pre-trained on large-scale Chinese corpora that may partially overlap with publicly available judicial documents, potential data exposure bias cannot be entirely excluded. This may further contribute to its comparatively strong baseline performance on the testing data. Overall, these experimental results verify the effectiveness of contextual patterns in capturing reasoning-related contextual signals within judicial texts.

**Table 5:** Performance comparison of full-text and pattern-based embeddings (^*^: Appendix B).

Method	Baseline	$CP_{top}^{Verb^{*}}$	$CP_{top}^{Modifier^{*}}$	$CP_{top}^{Function^{*}}$	Accuracy	Improvement
Baseline	BERT	–	–	–	84.60%	–
Full Text + Contextual patterns	BERT	15	20	30	89.51%	4.91%
		20	20	30	90.18%	5.58%
		30	30	40	89.64%	5.04%
		40	40	50	90.17%	5.57%
Baseline	RoBERTa	–	–	–	85.62%	–
Full Text + Contextual patterns	RoBERTa	15	20	30	90.45%	4.83%
		20	20	30	91.24%	5.62%
		30	30	40	90.18%	4.56%
		40	40	50	90.53%	4.91%
Baseline	TAIDE	–	–	–	92.35%	–
Full Text + Contextual patterns	TAIDE	15	20	30	91.45%	-0.90%
		20	20	30	92.40%	0.05%
		30	30	40	92.64%	0.29%
		40	40	50	92.52%	0.17%

To explore the potential of LLMs for inferring legal stances, we compared our proposed method with Gemini 3.0 Pro. A total of 100 fact descriptions were randomly selected as test samples. Inference is conducted in two stages. In the first stage, Gemini produces inference results—classifying whether a fact description strengthens, weakens, or remains neutral with respect to the sentence—using the following prompt: “Acting as a legal professional following the Criminal Code and Civil Law, please judge the following fact descriptions which may influence sentencing outcomes during homicide trials. Please reply: strengthen, weaken, or remain neutral to the sentence.” In the second stage, Gemini is provided with three examples of “strengthen” and three examples of “weaken” for few-shot prompting before conducting inference.

Table 6 shows that our proposed method, trained on thousands of judicial verdicts, achieves 90% accuracy, compared to 88% and 69% for Gemini 3.0 Pro with and without few-shot prompting, respectively. Notably, among the failures of Gemini 3.0 Pro, there are 23 instances where the model became entangled in lengthy arguments and failed to provide a final stance. With few-shot prompting, the advantages are demonstrated in two ways: first, it significantly enhances inference accuracy by 19%; and second, it resolves the issue where Gemini 3.0 Pro failed to provide an explicit stance when processing complex judicial texts. Overall, our frequency-based metric proves more robust and reliable in capturing reasoning-related contextual signals.

**Table 6:** Performance comparison of full text + patterns vs. Gemini vs. Gemini + few shot.

Method	True	False	Accuracy
Gemini 3.0 Pro	69	8 + 23	69%
Gemini 3.0 Pro with few-shot prompting	88	12	88%
Full Text + Contextual patterns	90	10	90%

#### 4.2.2 Knowledge graph construction

**Figure 4:**
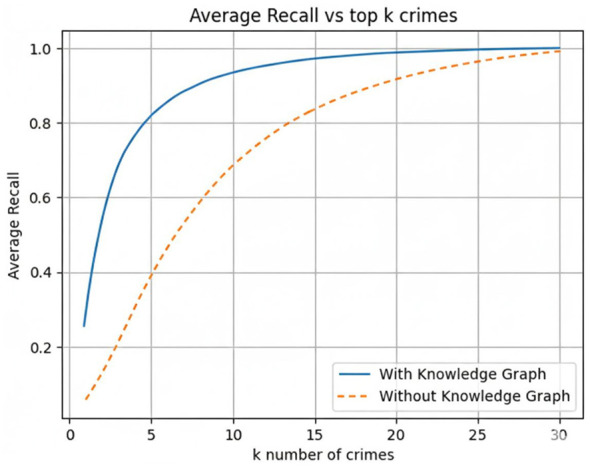
Comparison of average recall vs. top K offenses.

During knowledge graph construction, the graph structure is enhanced by incorporating additional context and cross-references. This enhancement ensures that the knowledge graphs capture hierarchical relationships among legal concepts, thereby improving semantic alignment accuracy. Table 7 presents the results following this graph enhancement. Taking Article 361 as an example, the penalty clause is absent in the original article text. After enhancement, Article 361 is cross-referenced with the three preceding articles, each of which defines a criminal condition and its corresponding penalty. This enhancement significantly strengthens the logical consistency and legal correctness of the model. Here, recall values are used to verify the effectiveness of the knowledge graphs; higher recall indicates that a greater number of law articles and clauses are accurately identified. Figure 4 illustrates the average recall performance across different top-K offenses. Our method consistently achieves higher recall values compared to the baseline models.

**Table 7:** Knowledge graph enhancement.

Article	Original length	After length	Growth rate
Article 100	85	131	54.11%
Article 101	67	146	117.91%
Article 102	33	101	206.06%
Article 103	75	99	32.00%
Article 104	74	96	29.72%
...	...	...	...
Article 346	94	179	90.42%
Article 351	31	36	16.13%
Article 353	87	98	12.64%
Article 361	33	173	424.24%
Article 362	66	313	374.24%

Furthermore, we compare the proposed framework with several baselines, including random, TF-IDF, BM25, BERT, RoBERTa, Longformer, Lawformer, and TAIDE. Performance is evaluated using the metrics: Priority Ranking Recall, Precision@2, Precision@6, Precision@10, and Rank Position Cost. The results in Table 8 demonstrate that our method significantly outperforms the baseline models across all evaluation metrics, achieving higher Priority Ranking Recall, Precision@2, Precision@6, and Precision@10 scores, while maintaining the lowest Rank Position Cost. Notably, among the baselines, lexical models such as TF-IDF and BM25 perform better than Transformer-based models like Lawformer and TAIDE. It suggests that lexical features are influential for identifying law articles. The comparison from a statistic perspective is elaborated in Appendix A. By incorporating cross-referential relationships and extracting corresponding key features, our method demonstrates its effectiveness through the accurate identification of relevant law articles.

**Table 8:** Performance comparison for all candidate law articles.

Method	Priority ranking recall	Precision @2	Precision @6	Precision @10	Rank position cost
Random	0.0393	0.0833	0.1389	0.0833	0.5897
TF-IDF	0.4938	0.4167	0.1667	0.1000	0.4662
BM25	0.4861	0.4167	0.1667	0.1000	0.4859
BERT	0.2568	0.1667	0.1389	0.0833	0.7408
RoBERTa	0.2573	0.1667	0.1389	0.0833	0.6826
Longformer	0.2789	0.1667	0.1389	0.1000	0.5813
Lawformer	0.2313	0.0833	0.1389	0.0833	0.6852
TAIDE	0.3759	0.1392	0.1065	0.0797	0.6820
Ours	0.6691	0.5602	0.2329	0.1551	0.1239

#### 4.2.3 Hierarchical architecture design

To address the challenge of distinguishing between similar legal provisions, hierarchical structures are designed. In legal practice, the reasoning process of judges is top-down, proceeding from criminal charges to law articles, clauses, and penalties. Similarly, hierarchical crime–law pair alignment follows the same path. In addition, a multi-granularity encoder is introduced to capture semantic meanings at multiple levels of abstraction. Table 9 demonstrates the performance comparison of our model with and without Hierarchical Architecture Design (HAD) across different law articles. The effectiveness of HAD in distinguishing similar law articles is thereby evaluated.

**Table 9:** Performance comparison w/o and with HAD^*^ across different law articles (^*^: Appendix B).

Article	Method	Priority ranking recall	Precision @2	Precision @6	Precision @10	Rank position cost
Article 174	Our w/o HAD	0.8547	0.8167	0.1473	0.1256	0.0355
	Our with HAD	**0.8847**	**0.8367**	**0.1673**	**0.1506**	**0.0055**
Article 177	Our w/o HAD	0.2375	0.5020	0.2310	0.1670	0.1016
	Our with HAD	**0.2575**	**0.5020**	**0.2510**	**0.1673**	**0.0516**
Article 200	Our w/o HAD	0.4009	0.2175	0.1637	0.1012	0.7112
	Our with HAD	**0.4343**	**0.5020**	**0.2231**	**0.1339**	**0.2092**
Article 276	Our w/o HAD	0.6391	0.3014	0.1278	0.0937	0.1102
	Our with HAD	**0.6633**	**0.3347**	**0.1422**	**0.1109**	**0.0887**
Article 161	Our w/o HAD	0.1253	0.5367	0.2426	0.1175	0.1908
	Our with HAD	**0.8794**	**0.5857**	**0.2510**	**0.1506**	**0.1305**
Article 309	Our w/o HAD	0.1220	0.5506	0.2857	0.1393	0.3388
	Our with HAD	**0.8952**	**0.6004**	**0.3626**	**0.2175**	**0.2578**
All	Our w/o HAD	0.3966	0.4875	0.1997	0.1241	0.2480
	Our with HAD	**0.6691**	**0.5602**	**0.2329**	**0.1551**	**0.1239**

Bold values represent the performance of our model with Hierarchical Architecture Design (HAD).

#### 4.2.4 Multi-perspective scoring

Multi-perspective scoring integrates dense representations, multi-vector analysis, and lexical retrieval to assess the semantic relationships between fact descriptions and law articles. This approach provides a comprehensive evaluation and improves alignment accuracy. Mean Reciprocal Rank (MRR) is employed to assess the ranking quality of our model and validate the effectiveness of the multi-perspective scoring mechanism.

MRR is defined as the average of the reciprocal ranks of the first relevant results for a set of queries, as Equation 12.

$$\begin{array}{c} \text{MRR}=\frac{1}{\left|Q\right|}\overset{\left|Q\right|}{\underset{i=1}{\sum }}\frac{1}{{\text{rank}}_{i}} \end{array}$$ (12)

where |*Q*| is the total number of fact descriptions, and rank_*i*_ is the ranking of the first relevant law article for the *i*-th description.

The results in Table 10 demonstrate that the multi-perspective scoring mechanism provides a more holistic assessment of relevant legal concepts compared to individual scoring components. Our full proposed framework, incorporating all scoring methods, achieves the highest MRR.

**Table 10:** Performance comparison of various multi-perspective scoring configurations across all candidate law articles.

Method	Priority ranking recall	Precision @2	Precision @6	Precision @10	Rank position cost	MRR
Dense	0.4913	0.3204	0.2050	0.1375	0.2290	0.28
Multi-vector	0.5084	0.3009	0.1780	0.1208	0.1751	0.28
Lexical	0.5030	0.3636	0.1929	0.1445	0.2973	0.33
Dense + Multi-vector	0.5326	0.3037	0.2045	0.1319	0.1786	0.31
Dense + Lexical	0.5309	0.3385	0.1934	0.1403	0.2268	0.33
Multi-vector + Lexical	0.5589	0.2451	0.1813	0.1286	0.1692	0.36
All	0.6692	0.5602	0.2329	0.1551	0.1239	1.00

Experimental results indicate that contextual patterns effectively capture reasoning-related contextual signals. The average recall of the knowledge-enhanced framework demonstrates a significant improvement over baselines lacking graph enhancement by addressing the challenge of complex statutory cross-references. Through the implementation of a hierarchical architecture for crime-law pairs and a multi-granularity encoder, the problem of high semantic similarity between fact descriptions and law articles is mitigated. Multi-perspective scoring further improves overall performance by refining ranking quality. Collectively, all components improve alignment accuracy while ensuring logical transparency and interpretability.

#### 4.2.5 Limitations and legal applicability

Although our proposed framework demonstrates superior performance in legal concept alignment through the above components, computational relevance is not equivalent to presenting legal reasoning. The distinctions between a computational-relevance-based alignment and a legally applicable system can be addressed across three aspects. First, our model computes textual similarities between fact descriptions and law articles, whereas legal professionals independently interpret the law and judge its normative relevance. Second, our model identifies contextual patterns as indicators of legal relevance, whereas legal experts utilize legal reasoning. Third, our model recommends candidate law articles through score calculation, whereas judges determine applicable provisions based on legally qualifying facts. Therefore, our proposed framework, AI-assisted legal concept alignment, can serve as a useful and interpretable tool that assists legal professionals during judicial processes rather than replacing legally grounded decision-making.

In this paper, legal domain experts are involved mainly in two ways. First, experienced lawyers contributed annotations of legal stances that serve as the ground truth for contextual pattern identification and provided consultation on legal perspectives. Second, sentencing outcomes handed down by judges are used to verify the effectiveness of legal concept alignment, while the experiments were conducted by the researchers. Furthermore, it is suggested that legal professionals be deeply involved in the practical application of this framework in the future to ensure legal applicability and interpretability.

## 5 Conclusion

In this paper, an AI-assisted legal concept alignment framework is proposed that capture reasoning-related contextual signals through contextual pattern identification and resolves statutory cross-references using law-centric knowledge graphs. Contextual patterns capture contextual indicators associated with legal reasoning from judicial texts and strengthen relevant signals. Law-centric knowledge graphs and hierarchical architecture designs collectively address statutory cross-references among legal elements and the semantic ambiguity inherent in highly related legal content. A multi-perspective scoring mechanism further enhances alignment accuracy by improving ranking quality. Experimental results show that the proposed framework captures both fine-grained details and broad semantic relations within legal content, achieving significant improvements in legal concept alignment compared to baseline methods while providing interpretability.

Future work may extend the applicability of this framework in two ways. First, although contextual pattern identification improves interpretability, the selection of patterns and distillation designs should be tailored to domain-specific legal terminology. Second, since the experiments were conducted primarily on criminal cases within Civil Law systems, and the knowledge graphs and alignment mechanisms are based on codified statutes, the framework could be adapted to common law systems or mixed jurisdictions by incorporating judicial precedents and case-based reasoning. Further exploration into these areas could provide deeper insights into the legal domain and enhance the generalizability of the framework.

Finally, this work is intended to support legal professionals rather than replace judicial decision-making. The framework focuses on facilitating legal concept alignment and ensuring interpretability based on computational relevance, as opposed to automating legal judgments. Moving forward, human-centric AI collaboration will be essential.

## Data Availability

Publicly available datasets were analyzed in this study. This data can be found here: Law & Regulation Database (https://law.moj.gov.tw/Eng/index.aspx) Open Data Platform (https://opendata.judicial.gov.tw/).

## Appendix A

To compare our proposed framework with baseline models from a statistical perspective, the Wilcoxon Signed-Rank Test is conducted. Table A1 in Appendix A shows that for all tested pairs, the *p*-values are less than 0.05. Specifically, our proposed framework demonstrates a significant performance difference compared to all baseline models. The extremely low *p*-values for Transformer-based models (BERT, RoBERTa, Longformer, Lawformer, TAIDE) indicate that our performance superiority is consistent across the dataset. The differences with classical methods like TF-IDF and BM25 (0.02 < *p* < 0.05), though significant, show a narrower margin of dominance.

**Table A 1:** Comparison by Wilcoxon Signed-Rank Test.

Method pair (*n* = 50)	Statistic	*P*-value
Ours vs. TF-IDF	324.5	0.02148
Ours vs. BM25	237.0	0.04680
Ours vs. BERT	70.0	2.1535e-06
Ours vs. RoBERTa	0	1.3422e-07
Ours vs. Longformer	35.5	5.7245e-07
Ours vs. Lawformer	69.0	1.3005e-06
Ours vs. TAIDE	0	1.3422e-07

## Appendix B

The table of notations used throughout the analysis is listed below Table A2.

**Table A 2:** Notations used in the analysis.

Symbol	Description	Table
$CP_{top}^{Verb}$	Contextual patterns comprise the most frequent contextual words categorized as verbs, based on their *rd* scores.	3,4,5
$CP_{top}^{Modifier}$	Contextual patterns comprise the most frequent contextual words categorized as modifiers, based on their *rd* scores.	3,4,5
$CP_{top}^{Function}$	Contextual patterns comprise the most frequent contextual words categorized as function words, based on their *rd* scores.	3,4,5
Our w/o HAD	Our model without Hierarchical Architecture Design.	9
Our with HAD	Our model with Hierarchical Architecture Design.	9
